# Local Anesthesia Approach for Percutaneous Screw Fixation of an Impacted Subcapital Femoral Neck Fracture: A Technique for High-Risk Patients

**DOI:** 10.7759/cureus.38532

**Published:** 2023-05-04

**Authors:** Eric B Smith, Gregory K Deirmengian

**Affiliations:** 1 Orthopaedic Surgery, Rothman Orthopaedic Institute, Philadelphia, USA

**Keywords:** hip fracture, femoral neck fracture, bupivacaine, local anesthesia, anesthesia

## Abstract

Elderly, frail patients and those who have substantial medical co-morbidities who sustain hip fractures present a challenging problem for treatment as they are at very high risk for complications from surgical intervention. The functional outcomes, pain levels, and mortality rates all worsen when non-surgical treatment is used. The safety of administering general or spinal anesthesia may be a concern in certain cases. Other modalities, such as epidural or caudal anesthesia, may be an option; however, the use of local anesthesia may be advantageous for patients with non-displaced and impacted femoral neck fractures undergoing surgical intervention. We present a case report describing the successful treatment of an elderly male who had relative contraindications to spinal anesthesia and high risk for general anesthesia and was successfully treated with percutaneous screw fixation of a femoral neck fracture using local anesthesia with a light, monitored anesthetic.

## Introduction

There are approximately 300,000 hip fractures in patients over 65 years of age occurring in the United States each year [1]. Like all fractures in the hip, rapid treatment is beneficial to allow for early mobilization and fewer complications. Impacted and non-displaced fractures of the femoral neck may require more urgent treatment with screw fixation; otherwise, displacement can occur that would require treatment with arthroplasty [2]. This leads to increased risks, including longer surgical time, increased blood loss, and the inherent risks of periprosthetic infection and dislocation that are not seen with closed fixation and percutaneous pinning.

Patients undergoing these procedures may be elderly and frail with substantial medical co-morbidities. Often spinal anesthesia can be performed, however, there may be contraindications such as coagulopathy, aortic stenosis, increased intracranial pressure, severe arthritic degeneration, or spinal fusion that limit the ability to perform the procedure [3,4]. There are high risks with general anesthesia in patients with significant heart disease and poor pulmonary function. Also, medications administered during general anesthesia may have detrimental effects on elderly patients, such as delirium, nausea and vomiting, and urinary retention [5]. Some patients may have a combination of co-morbidities that make them so risky for anesthesia that a decision needs to be made whether or not to operate. Unfortunately, these patients are often treated non-surgically, with a 30-day mortality rate of 65% or higher [6,7]. Therefore, for patients with contraindications for spinal anesthesia and deemed too ill to receive general anesthesia, a technique for simple local infiltration to the surgical site would be ideal.

There is information in the literature regarding regional blocks, but these blocks may provide incomplete analgesia to the areas that generate pain from the fracture or surgical technique and may risk nerve injury [8-10]. Historically, textbooks have described the use of local anesthesia for these procedures [11]; however, the teaching and knowledge of this option over the years may have been lost. Recently, two small studies from Australia and China discussed local anesthetic infiltration during these surgeries. One describes the surgery using a small incision [12] and the other [13] describes percutaneous fixation with two screws. This study presents a surgical instructional technique for percutaneous fixation with three cannulated screws in an elderly male patient with substantial medical co-morbidities. The procedure was performed successfully utilizing only local anesthesia with light, monitored anesthetic.

## Case presentation

The patient

This case report describes the procedure on a 97-year-old male walker-assisted ambulatory who fell and sustained a right valgus impacted subcapital femoral neck fracture. Written informed consent was obtained by the patient’s daughter and power of attorney to participate in this case report and authorize publication. His past medical history was significant for dementia, a severe asbestos-related pulmonary disease requiring oxygen, aortic stenosis, arteriosclerotic heart disease, Mobitz type 1 atrioventricular (AV) block, and bradycardia (previously refused pacemaker), hypertension and hyperlipidemia. He was rated American Society of Anesthesiologists Physical Status (ASA-PS)4. He was in severe pain with his injury and the family requested surgery if at all possible. Spinal anesthesia was contraindicated due to severe aortic stenosis and general anesthesia was considered dangerous due to his pulmonary disease and concern the anesthetic agents would worsen the AV conduction block. The patient was cleared for surgery to be performed using local anesthesia and light, monitored sedation.

The technique

**Figure 1:**
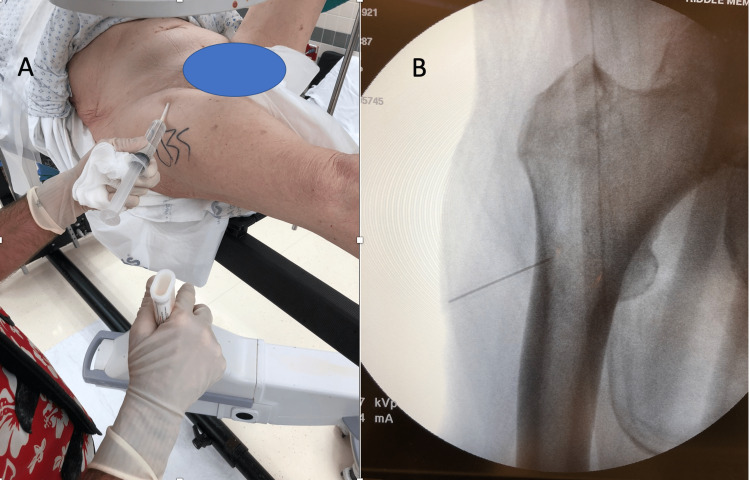
Localizing the trajectory and position of the femoral neck utilizing AP fluoroscopy AP: Anterior-posterior (A) Grossly assess the level prior to prepping the skin by placing the needle anterior on the proximal femur. (B) Fluoroscopic image visualizing the needle at the level of the lesser trochanter overlying the lateral cortex.

The patient was brought into the operating room on a stretcher. Upon arrival, he received 2g of cefazolin as antibiotic prophylaxis, 25mg of ketamine (0.42mg/kg for this 60kg patient, which is less than half of a typical dose), and 0.5mg of midazolam (8.3mcg/kg which is about half of the typical lowest dose) prior to being moved from the stretcher to the fracture table. He was carefully moved onto the fracture table and all limbs were carefully secured in position. Anterior-posterior (AP) fluoroscopy was used to confirm positioning and ensure no displacement of the fracture. After time out and prior to prepping, a syringe filled with 30cc 0.5% bupivacaine without epinephrine and a capped 1.5-inch 22-gauge needle was placed over the skin on the anterior aspect of the hip and fluoroscopy was used to localize the trajectory and position of the femoral neck (Figure 1).

**Figure 2:**
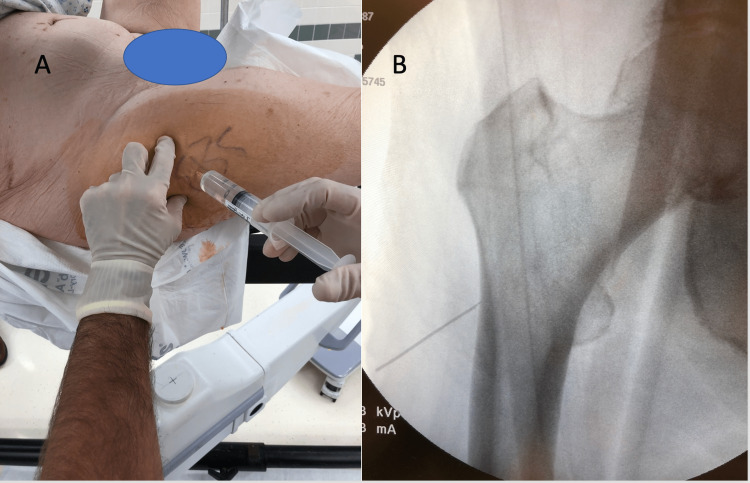
Local anesthesia application procedure images (A) After prepping the site, the skin, subcutaneous, and fatty layers were infiltrated with local anesthetic under sterile technique in the areas where skin incisions will be made. The insertion site was determined with fluoroscopy, and the anterior and posterior borders of the femur were palpated with the contralateral hand. (B) The needle was advanced under fluoroscopic view to the planned insertion site of the drill pins on the lateral cortex of the femur, beginning inferiorly and progressively moving proximally to infiltrate the entire periosteum of the targeted lateral femur.  A 1.5-inch 22 gauge needle was used in this case.

ChloraPrep™ (a broad-spectrum antiseptic) was used to prepare the area and then local infiltration of the site was performed under sterile conditions. The gloved contralateral hand was used to palpate the anterior and posterior aspects of the femur. Skin, subcutaneous, and fatty layers were infiltrated with the local anesthetic in the areas where skin incisions would be made (Figure 2A). The insertion site was determined with fluoroscopy and the needle was advanced under fluoroscopic guidance to the planned insertion site of the drill pins on the lateral cortex of the femur. The needle was placed at the inferior guide pin site initially, and infiltration of the periosteum began, moving progressively proximally to infiltrate the entire periosteum of the targeted lateral femur. A 1.5-inch 22-gauge needle was used in this case, but for larger patients, a spinal needle may be necessary to reach the lateral cortex (Figure 2B). Approximately one-third to one-half of the bupivacaine was infiltrated into the soft tissues and the remainder onto the periosteum of the lateral femoral cortex. For larger patients, additional amounts of bupivacaine can be utilized, though the maximum single dose is typically 175mg.

**Figure 3:**
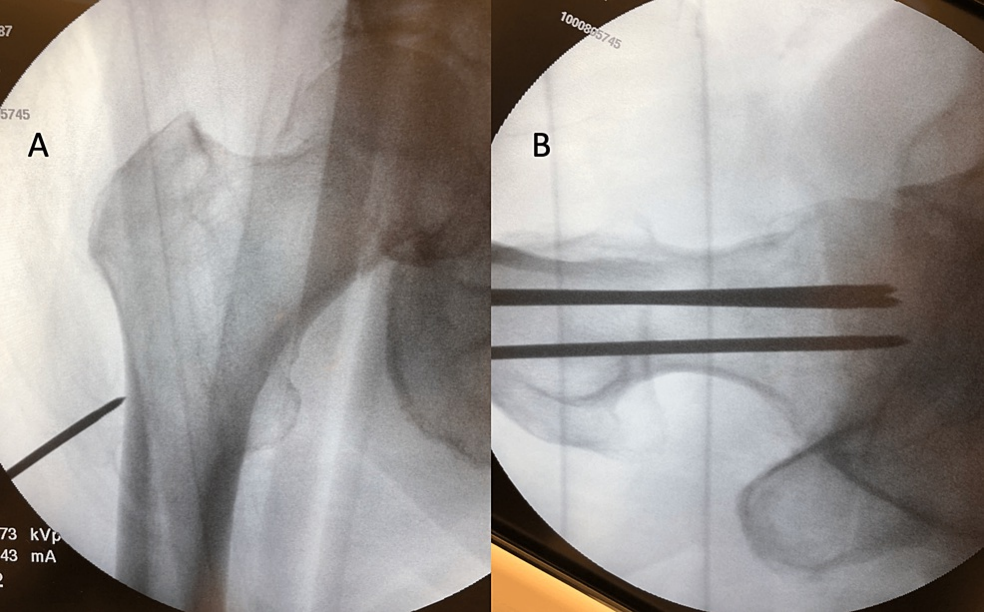
Treatment site images after preparation After scrubbing, gowning, prepping, and draping, plenty of time should have elapsed to allow for the full anesthetic effect. (A) Fluoroscopic AP image showing guide pin placed percutaneously through the skin onto the lateral cortex at the level of the lesser trochanter. (B) Lateral fluoroscopic image after all three pins were placed percutaneously in a triangular and parallel fashion.

After infiltration of the bupivacaine, the surgeon performed a routine hand scrub and was gowned. The skin was prepped again with ChloraPrep™, and the site was draped in a sterile fashion. The injection was performed prior to this, allowing plenty of time for the anesthetic to take effect. A guide pin was used to pierce through the skin at the appropriate level and positioned at the lateral cortex no lower than the level of the lesser trochanter. It was drilled through the bone, and positioning was confirmed on the AP and lateral fluoroscopic views. Two additional guide pins were passed in a similar fashion proximally - one slightly anterior and the other slightly posterior creating a triangular, parallel configuration (Figure 3).

**Figure 4:**
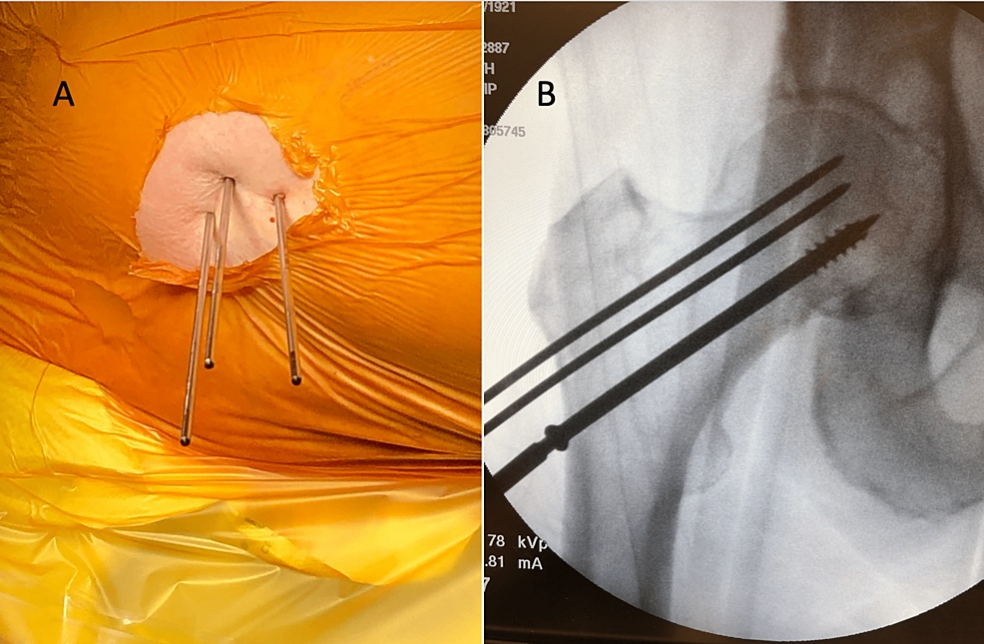
Intraoperative images (A) Gross visualization of three pins placed percutaneously in a triangular, parallel fashion. Small incisions were then made in the skin and the guide pins were measured. A cannulated drill was used over each guide pin to drill the lateral cortex, and screws were then inserted over each guide pin. (B) Fluoroscopic anterior-posterior (AP) image showing placement of the inferior 7.3 cannulated screw over the guide pin.

Once all three pins were in place, a small incision through the skin at each pin site was made with a scalpel. Each pin was measured; the lateral cortex was drilled over each guide pin with a cannulated drill bit, and three 7.3mm cannulated screws were inserted (Figure 4).

**Figure 5:**
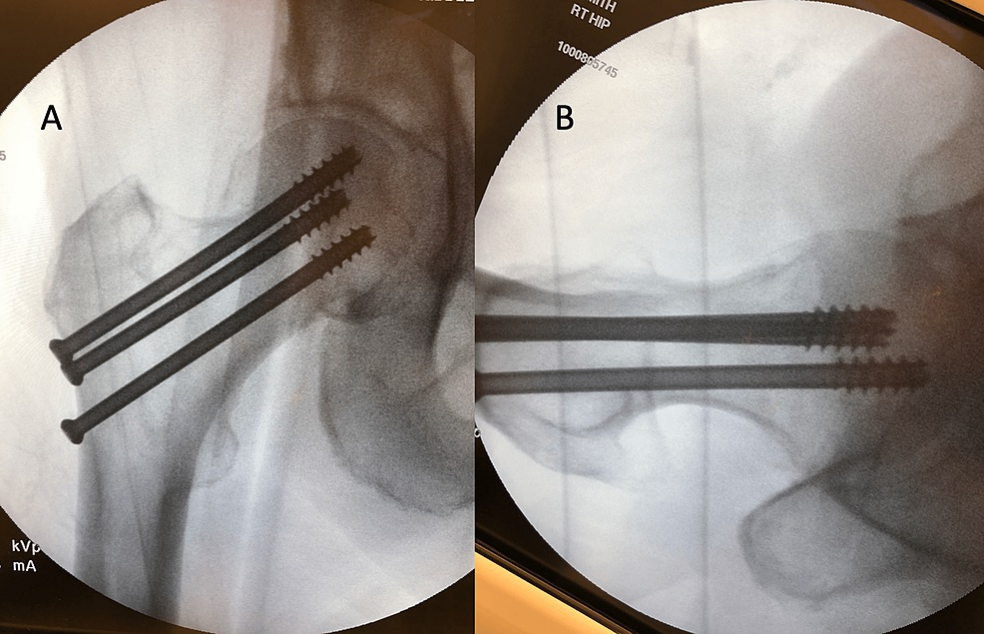
Post-operative images (A) Anterior-posterior (AP) and (B) lateral fluoroscopic images showing screw placement after guide pin removal.

**Figure 6:**
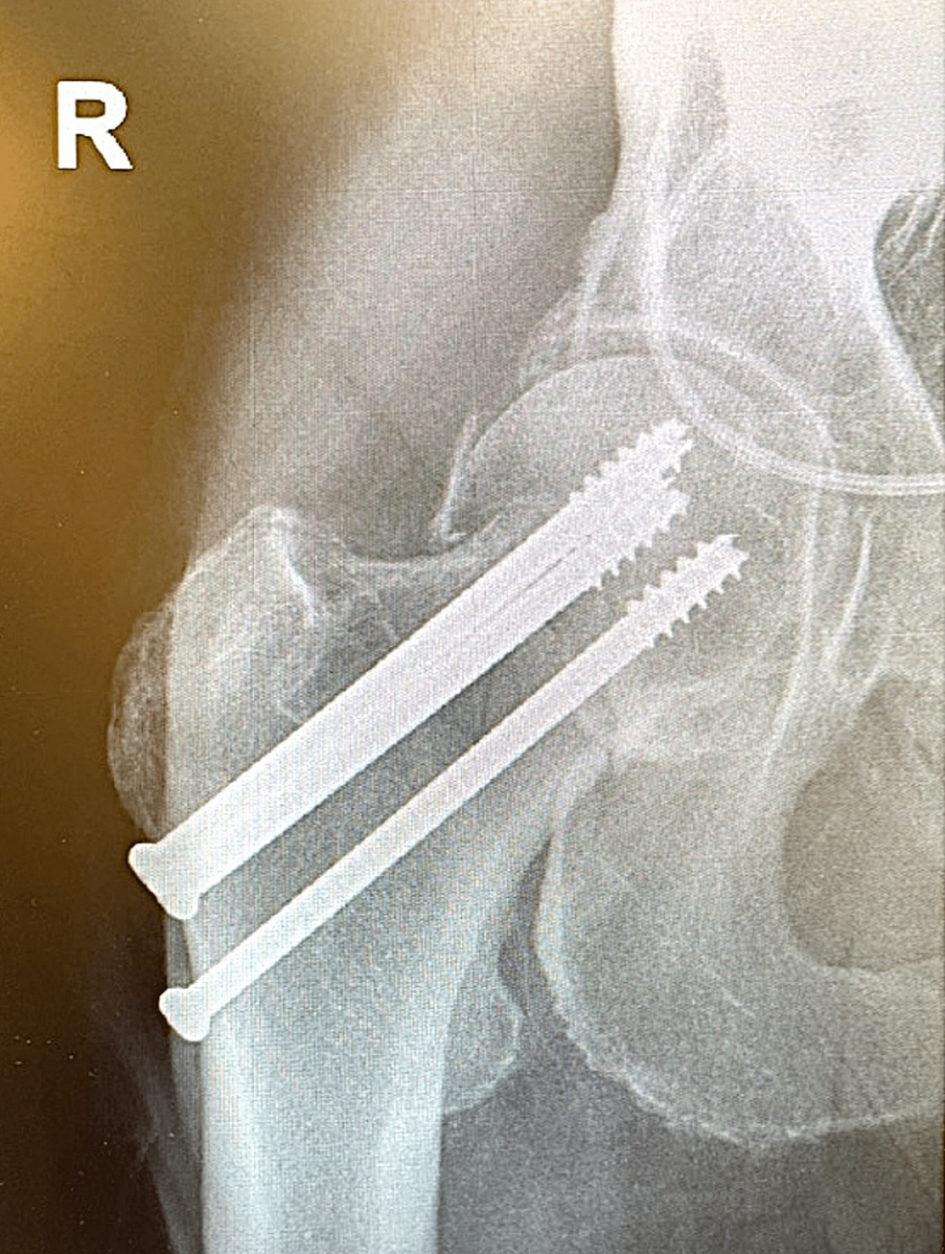
Three weeks post-operative AP radiograph Anterior-posterior (AP) radiograph of the right hip taken three weeks post-operatively shows no displacement or loss of fixation.

The position of each was confirmed with fluoroscopy, and the guide pins were then removed (Figure 5). The final AP and the lateral fluoroscopic and subsequent three-week post-operative radiograph showed a good position of the implants and no displacement of the fracture (Figure 6).

The three incision sites were each closed with a single 2.0 vicryl subcutaneous stitch and glue for the skin edges. An occlusive dressing was applied. Upon arrival at the recovery room, the patient received a single dose of 25mcg fentanyl (0.42mcg/kg). The surgical time from incision to dressing application for the procedure was 20 minutes and the overall room time was less than one hour. The patient remained medically stable during the procedure, continuing his pre-existing 4 liters of nasal cannula oxygen. At no time point did his oxygen saturation drop below 95%. His heart rate was approximately 100 beats per minute (bpm) upon entering the room; increased to 110 bpm during positioning on the fracture table; remained 80-110 bpm during the surgical procedure; and was 80 bpm upon arrival to the recovery room. Likewise, his blood pressure was 155/65 mm Hg upon entering the room; increased to 175/90 mm Hg during movement onto the fracture table; remained approximately 150/70 mm Hg during the surgery; and was 130/60 mm Hg upon arrival to the recovery room. His pre-operative hemoglobin was 13.3 and this decreased minimally to 12.4 on post-operative day two.

## Discussion

This case study shows a technique to perform percutaneous fixation of impacted or non-displaced femoral neck fractures using only 30cc of 0.5% bupivacaine locally as anesthesia. By infiltrating the nerve supply in the skin, soft tissues, and periosteum of the bone, successful local anesthesia is all that may be necessary for these procedures. Neither general nor spinal anesthesia is necessary in these instances. In this case, only tiny doses (less than half of the standard lowest doses of ketamine, midazolam, and fentanyl) of adjunctive medications were given for patient comfort.

Non-displaced and impacted femoral neck fractures should be surgically treated to avoid displacement and the need for more extensive surgeries (arthroplasty) [2]. With fixation, the patient can be mobilized immediately and begin weight bearing. It is well documented that early surgical treatment of hip fractures leads to lower complications and fewer mortality rates (5.8% 30-day mortality versus 6.5% if surgically treated after 24 hours) [1]. When elderly patients with hip fractures are treated non-operatively, one-third of patients will die within 30 days and during this time, pain can be difficult to manage as any movement of the fracture can be excruciating [6,7]. Additionally, these patients are typically placed on bedrest and, therefore, can develop bed sores and pulmonary complications and have very poor hygiene.

Other approaches have been reported to address this issue, including anesthetic blocks of the femoral nerve, fascia iliaca compartment, and quadratus lumborum; however, these either required increased dosing of narcotics during surgery or were in addition to general anesthesia [8-10]. Direct infiltration with local anesthetic has been previously described in Australia [12] and China [13] with good success. A recent study shows that surgical fixation of intertrochanteric hip fractures may be possible using soft tissue infiltration with local anesthesia along with monitored anesthesia care (MAC) [14]. Performing these surgeries under local anesthesia obviates the need for general and spinal anesthesia in patients that are too high risk or have contraindications to these procedures. This may reduce the intra and post-operative risks related to these modalities and may allow these high-risk patients to undergo surgery and avoid the complications associated with non-operative treatment.

## Conclusions

We performed a successful percutaneous fixation of an impacted valgus femoral neck fracture in an elderly male with significant medical comorbidities who otherwise would not have been cleared for anesthesia. This percutaneous technique with fluoroscopy allows for fixation with minimal trauma to the local tissue and does not disrupt the blood supply to the fracture site. Infiltration of local anesthesia provides sufficient anesthesia to the area so the patient can undergo surgery without undo stress. Performing percutaneous fixation of non-displaced and valgus-impacted femoral neck fractures in high-risk patients may be performed utilizing local anesthetic with light, monitored sedation.
